# Supplementary material for: ABE-ultramax for high-efficiency biallelic adenine base editing in zebrafish
Source: Nat Commun. 2024 Jul 4;15:5613. doi: 10.1038/s41467-024-49943-1 (PMC11224239; doi:10.1038/s41467-024-49943-1)
Supplement: Supplementary file 1 — Supplementary Information [file 41467_2024_49943_MOESM1_ESM.pdf]

Supplementary Information

ABE-Ultramax for high-efficiency biallelic adenine base editing in zebrafish

Wei Qin[1†], Fang Liang[2†], Sheng-Jia Lin[1], Cassidy Petree[1], Kevin Huang[1], Yu Zhang[1], Lin Li[3], Pratishtha Varshney[1], Philippe Mourrain[4], Yanmei Liu[3, 5*], and Gaurav K. Varshney[1*]

1. Genes & Human Disease Research Program, Oklahoma Medical Research Foundation, Oklahoma City, OK, USA.
2. Institute of Modern Aquaculture Science and Engineering, School of Life Sciences, South China Normal University, Guangzhou, Guangdong, 510631, China.
3. Key Laboratory of Brain, Cognition and Education Sciences, Ministry of Education, South China Normal University, 510631 Guangzhou, China.
4. Department of Psychiatry and Behavioral Sciences, Stanford University, Stanford, CA, USA.
5. Institute for Brain Research and Rehabilitation, and Guangdong Key Laboratory of Mental Health and Cognitive Science, South China Normal University, 510631 Guangzhou, China.

Supplementary Figures

a

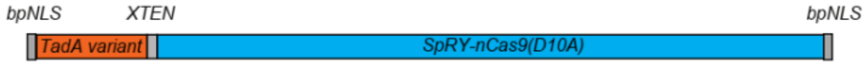

b

Position	23	27	36	47	48	51	76	82	84	106	108	109	111	119	122	123	127	146	147	149	152	154	155	156	157	166	167
WT TadA	W	E	H	R	P	R	I	V	L	A	D	A	T	D	H	H	N	S	D	F	R	Q	E	I	K	T	D
TadA7.10	R		L		A	L			F	V	N					Y		C	Y		P		V	F	N		
TadA8.17	R		L		A	L		S	F	V	N					Y		C	Y		P	R	V	F	N		
TadA8.20	R		L		A	L	Y	S	F	V	N							C	R		P	R	V	F	N		
TadA8e	R		L		A	L			F	V	N	S	R	N	N	Y		C		Y	P		V	F	N	I	N
TadA-8eKR	R		L		A	L			F	V	N	S	R	N	N	Y	K	C		Y	P	R	V	F	N	I	N
TadA-9e	R		L		A	L			F	V	N	S		N	N	Y	K	C		Y	P	R	V	F	N	I	N
variant1	R		L		A	L		S	F	V	N	S	R	N	N	Y		C		Y	P		V	F	N	I	N
variant2	R		L		A	L			F	V	N	S	R	N	N	Y		C		Y	P	R	V	F	N	I	N
variant3	R		L		A	L		S	F	V	N	S	R	N	N	Y		C		Y	P	R	V	F	N	I	N
variant4	R		L		A	L		S	F	V	N	S	R	N	N	Y		C	R	Y	P	R	V	F	N	I	N
variant5	R		L		A	L		S	F	V	N	S	R	N	N			C		Y	P	R	V	F	N	I	N
variant6	R		L		A	L		S	F	V	N	S	R	N	N	Y	K	C		Y	P	R	V	F	N	I	N
variant7	R		L		A	L			F	V	N	S		N	N	Y	K	C		Y	P	R	V	F	N	I	N
variant8	R		L		A	L	Y	S	F	V	N	S	R	N	N	Y		C		Y	P	R	V	F	N	I	N
variant9	R		L		A	L		S	F	V	N	S		N	N	Y	K	C		Y	P	R	V	F	N	I	N
variant10	R		L		A	L	Y	S	F	V	N	S	R	N	N			C	R	Y	P	R	V	F	N	I	N

Supplementary Figure 1. Genotypes of Reported and New TadA Variants

(a) Schematic diagrams of adenine base editors used in this study. XTEN represents a peptide linker composed of the amino acid sequence SGSETPGTSESATPES, which bridges the nucleoside deaminase domain and the nCas9 domain. bpNLS denotes bipartite nuclear localization signals.

(b) Mutations in TadA7.10 are colored in gray. Additional mutations reported in TadA8.17, TadA8.20, TadA8e, TadA8eKR, and TadA9e are colored in orange, green, cyan, pink, and purple, respectively. Based on TadA8e, all mutations from our 10 TadA variants are labeled in red.

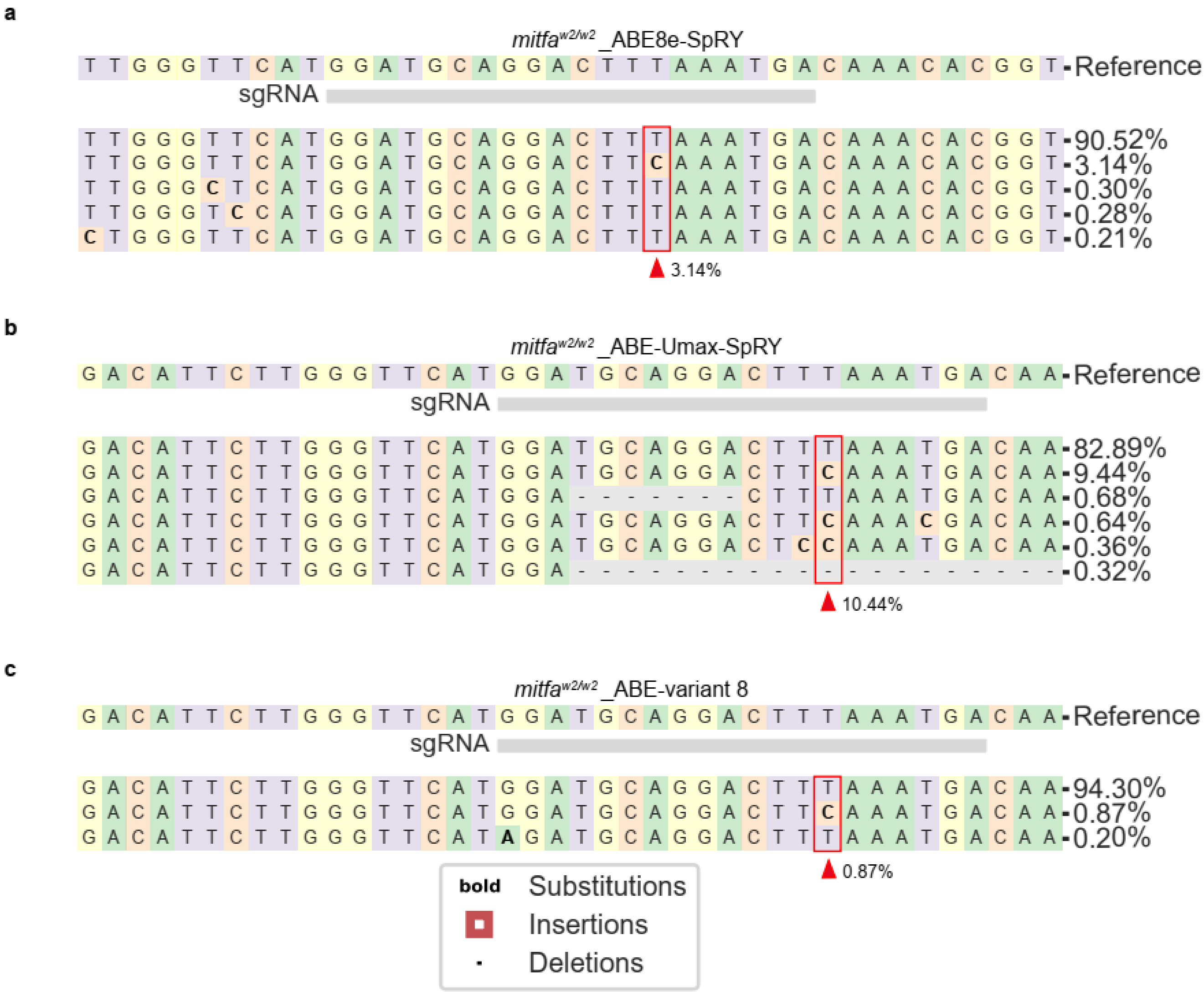

Supplementary Figure 2. On-Target Analysis of ABE8e-SpRY, ABE-Umax-SpRY, and Variant 8 Induced Base Conversion at the Mitfa Site Using Next-Generation Sequencing.
Genomic DNA was extracted from 30 injected embryos randomly selected from each group. After PCR fragment purification, the samples were subjected to paired-end sequencing using the MiSeq platform (Illumina). The sequencing data were analyzed using CRISPResso2. The desired T to C (A to G) conversion is highlighted in a red box with a red arrowhead on *mitfa*$^{w2/w2}$ locus (a) using ABE8e-SpRY (b)ABE-Umax-SpRY (c)ABE-variants 8

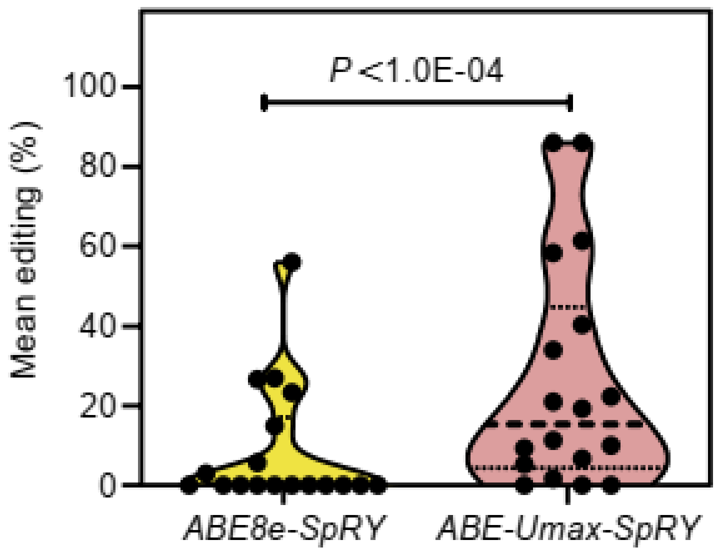

Supplementary Figure 3. Assessment of Mean Editing Efficiency of ABE8e-SpRY and ABE-Umax-SpRY

A comprehensive assessment of adenine base editing efficiency for ABE8e-SpRY and ABE-Umax-SpRY based on data from Figure 1 d and e. Each data point signifies the average editing activity at a specific site. The central dotted line indicates the mean of all data points. P-values are displayed at the top of the violin diagram. Wilcoxon Matched Pairs Signed Rank Test was performed (with P-values marked). Source data are provided as a Source Data file.

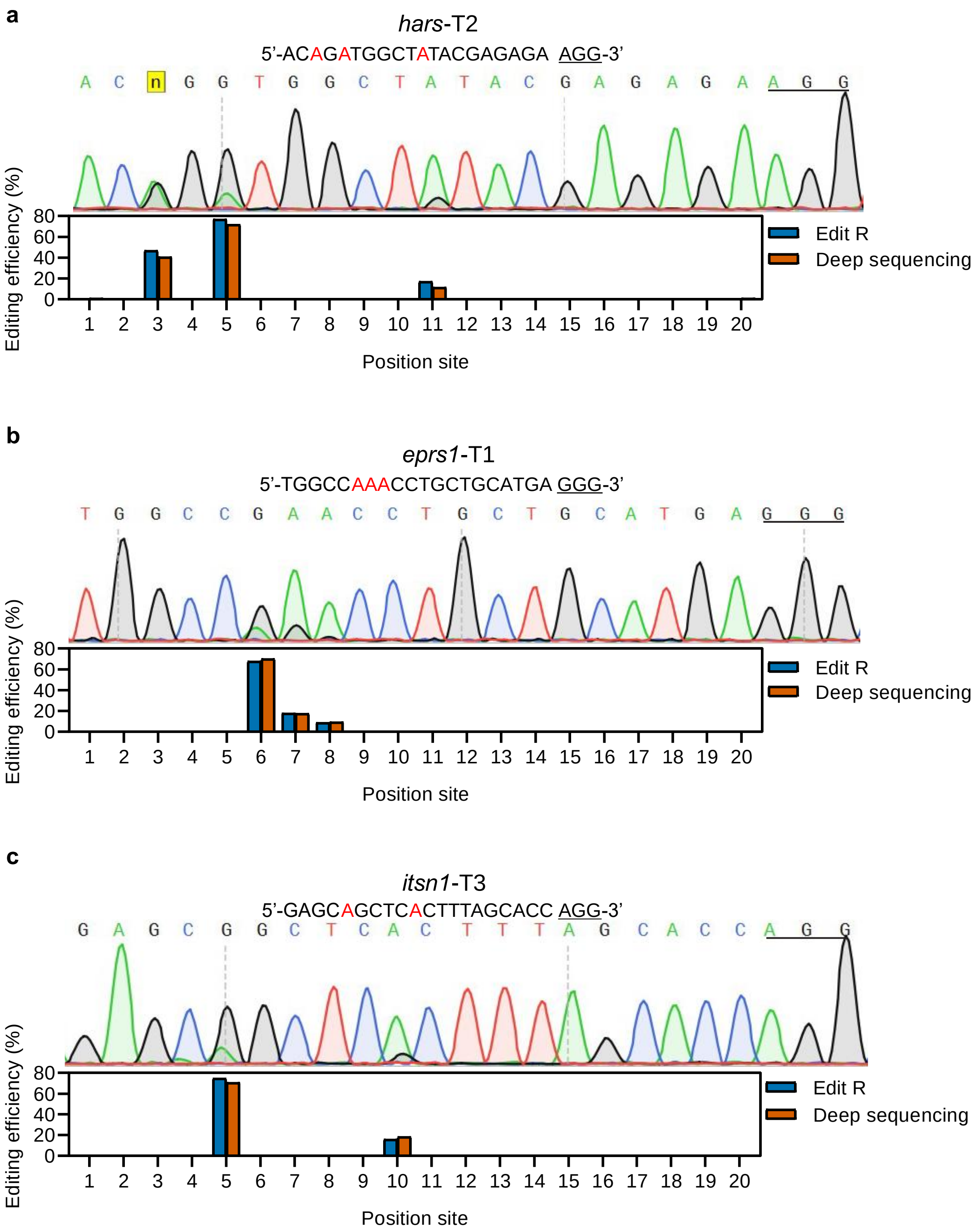

Supplementary Figure 4. Comparison of Editing Efficiency at the Same Locus by EditR and MiSeq Sequencing Analysis

Comparison was made for three different targets/genes: (a) *hars1*, (b) *eprs1*, and (c) *itsn1*. The PAM sequence is underlined. The targeted nucleotide is highlighted in red. Source data are provided as a Source Data file.

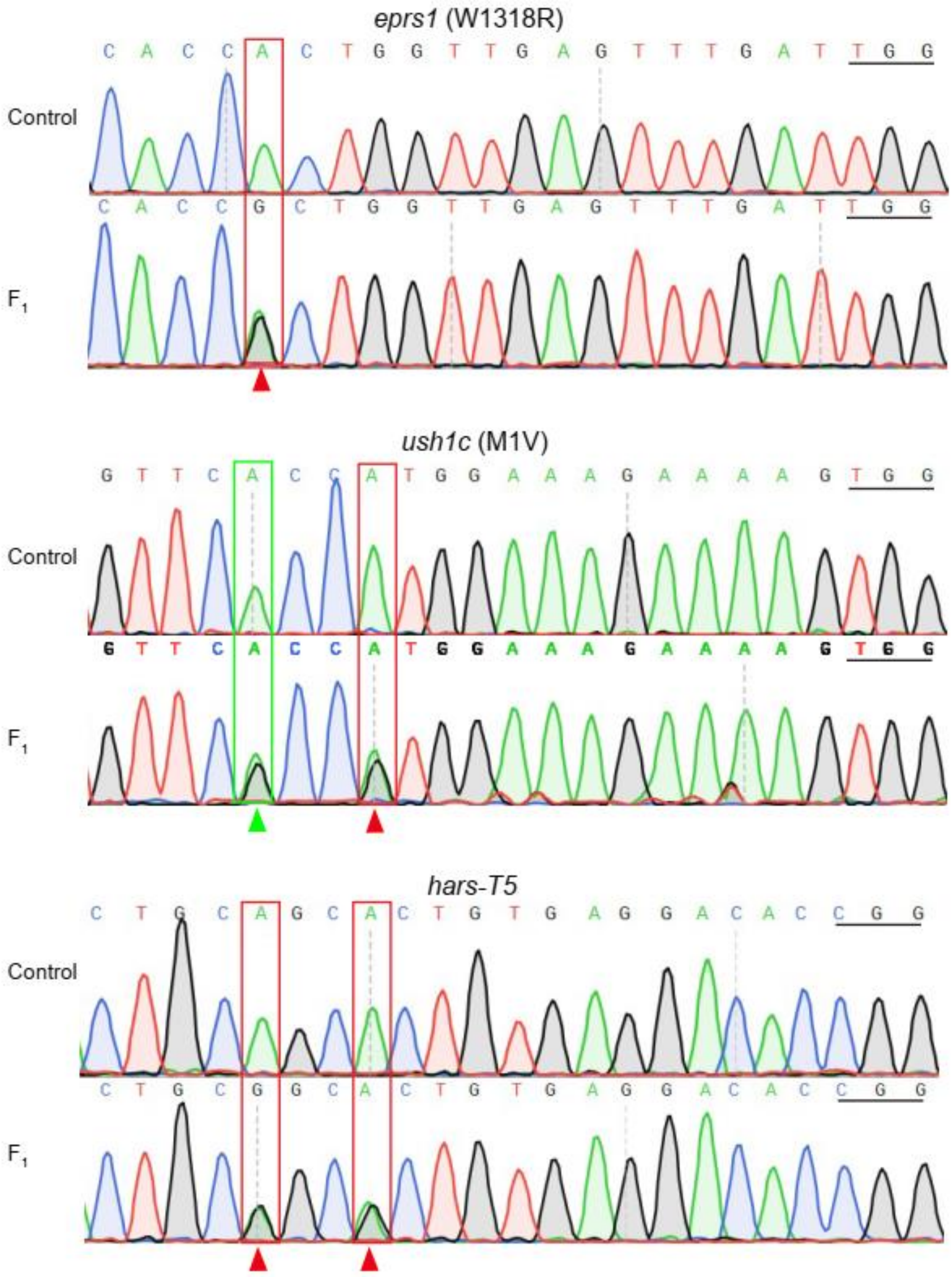

Supplementary Figure 5. Sanger sequencing results of *eprs1* (W1318R), *ush1c* (M1V) and *hars*-T5 F_1 embryos induced by ABE-Umax-nanos1.

The targeted sequence is shown with the PAM underlined. Red arrowhead indicates the expected nucleotide substitutions and the bystander base substitutions are indicated by a green arrowhead in the Sanger sequencing chromatograms.

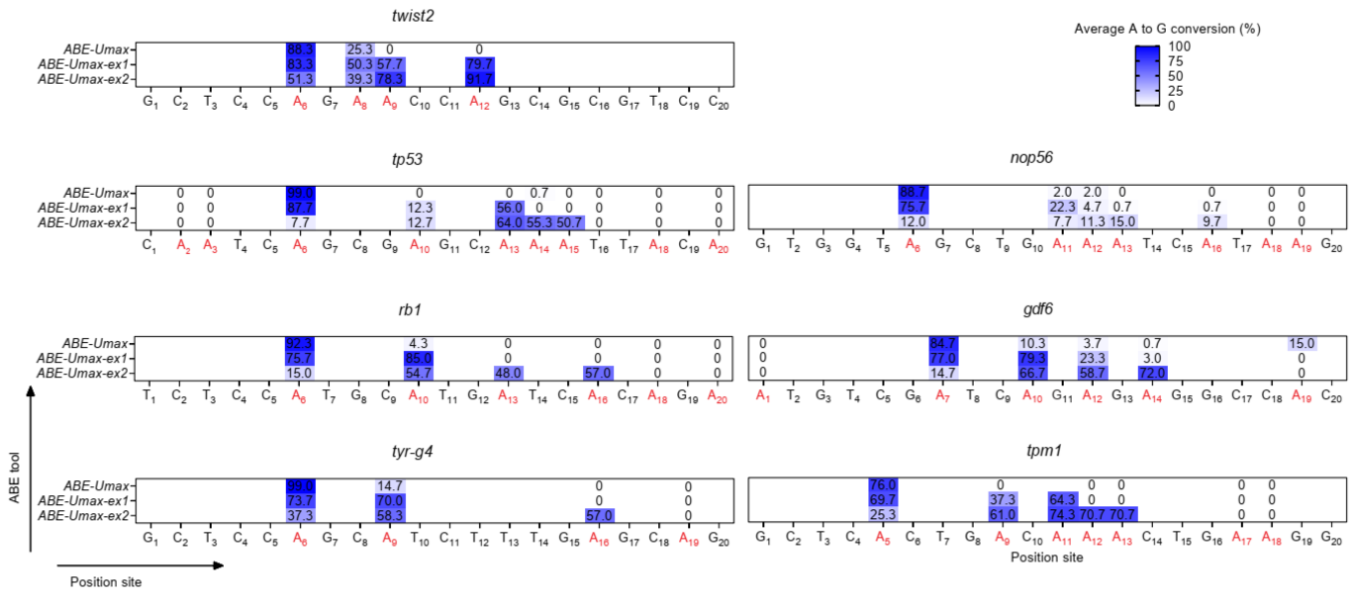

Supplementary Figure 6. Assessment of Efficiency and Targeting Window of ABE-Umax-ex1 and ABE-Umax-ex2

A-G conversion frequencies at each adenine nucleotide in the 20 bp protospacer were quantified using EditR. Three independent experiments were performed, and editing frequencies >0.20 were labeled in the heat map. The color mapping from blue to white represents the editing efficiency from 100% to 0%. Source data are provided as a Source Data file.

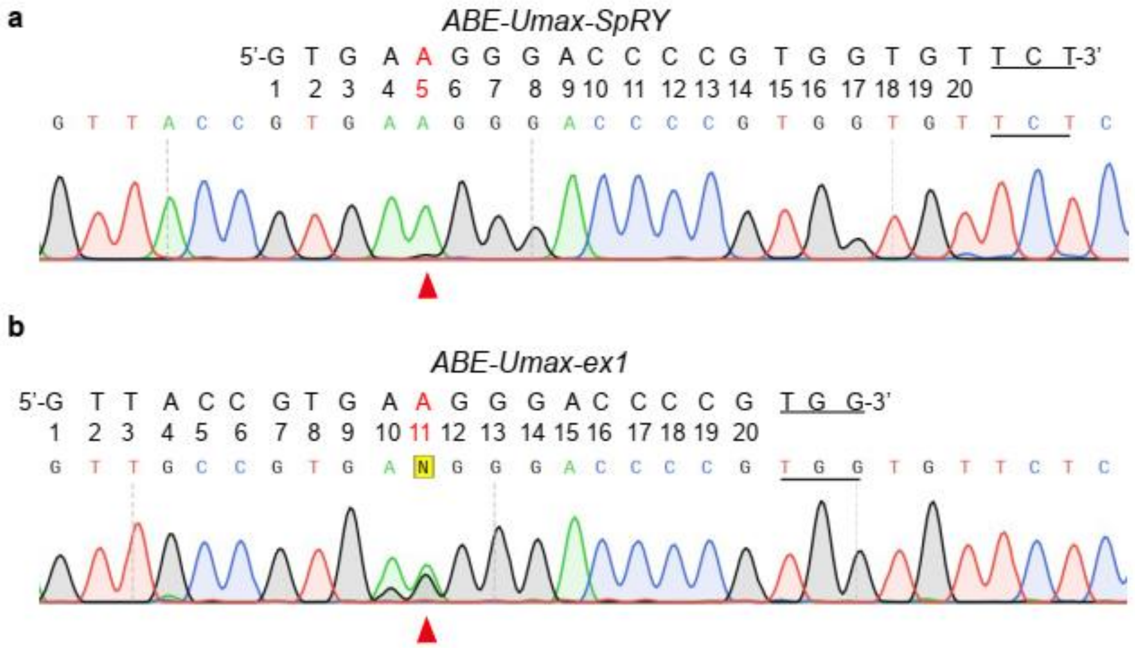

Supplementary Figure 7. Comparison of Editing Efficiency Between ABE-Umax-ex1 and ABE-Umax-SpRY at the Rpl9 Locus

The PAM sequence is underlined, and the targeted nucleotide is highlighted in red. Nucleotide substitutions (overlapping peaks) are indicated by a red arrowhead in the Sanger sequencing chromatograms. Comparison of (a) ABE-Umax-SpRY and (b) ABE-Umax-ex1 is shown at the same locus.

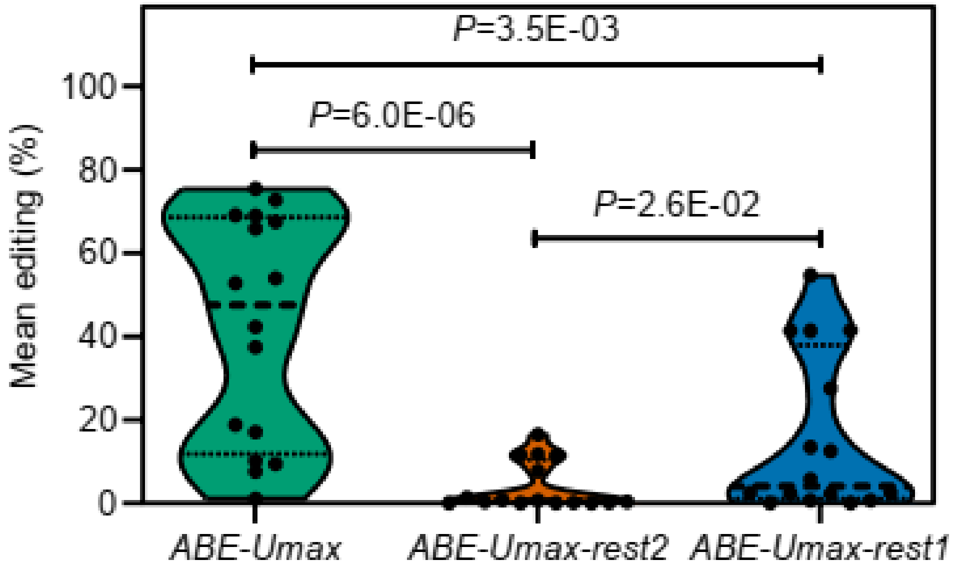

Supplementary Figure 8. Comprehensive Analysis of Adenine Base Editing Efficiency for ABE-Umax, ABE-Umax-rest2, and ABE-Umax-rest1

Each data point represents the average editing activity at a particular site. The central dotted line indicates the mean of all data points. P-values are marked at the top of the violin diagram. Two-tailed paired multiple t-tests were performed (with P-values marked). Source data are provided as a Source Data file.

a

Sample	Off-target site	Mismatched bases (bp)	Genome location	Sequence
tp53	1	1	24:26468287-26468309	CAATCAGgGAGCAAATTACA GGG
	2	2	19:8750173-8750195	CAATCAGgGtGCAAATTACA GGG
	3	3	4:18116204-18116226	tAATCAGaGtGCAAATTACA AGG

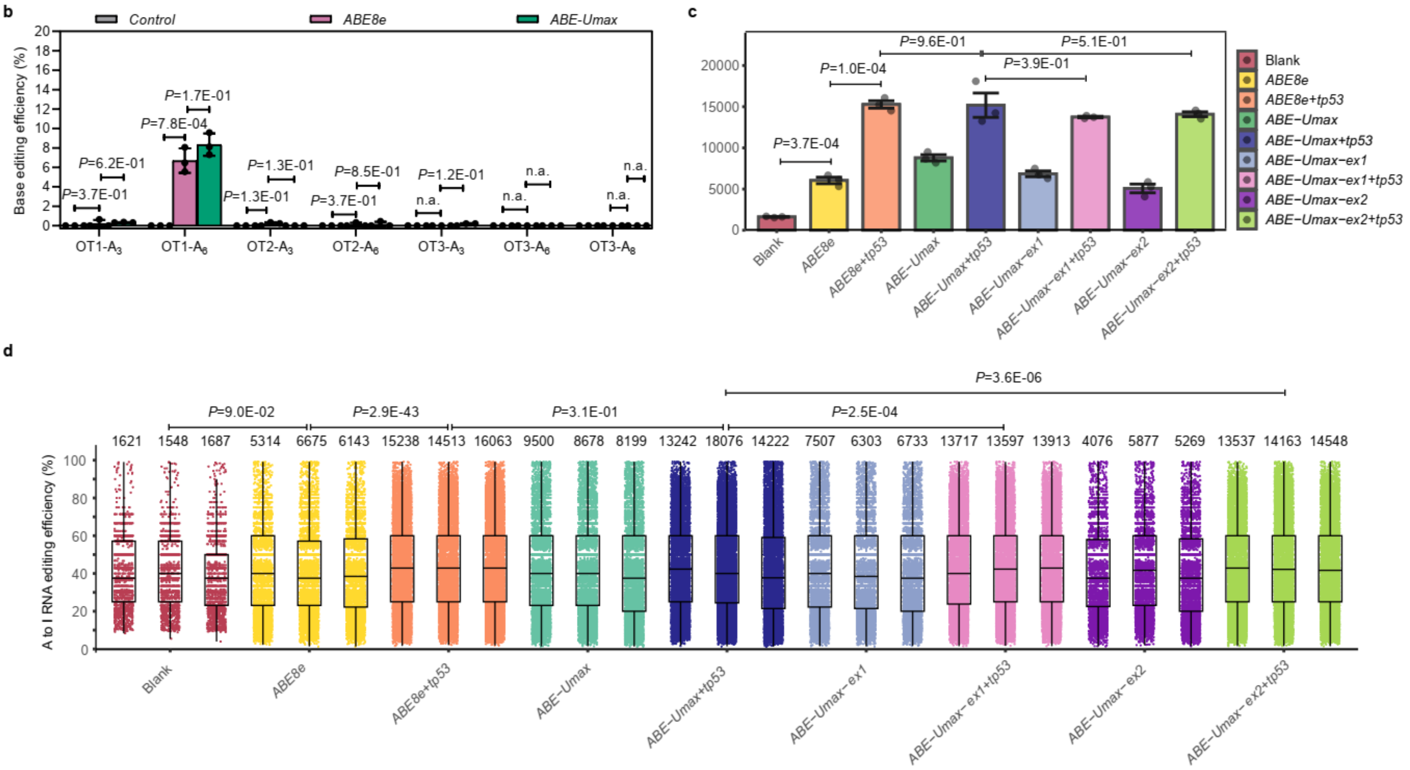

Supplementary Figure 9. Off-Target Analysis of ABE-Umax-Related Tools at the Tp53 Locus

(a) Information on the three most likely off-target sites (top three high-scoring sites) at the Tp53 locus. The mismatch bases in potential off-target sites are indicated in lowercase, and the potentially editable adenine is highlighted in red.

(b) DNA off-target analysis comparing ABE8e and ABE-Umax at the Tp53 locus. Editing efficiencies are shown. Error bars indicate mean ± s.d. (n = 3 biological replicates). Two-tailed paired t-tests were performed (with P-values marked).

(c) Transcriptome analysis showing the number of edited adenine nucleotides in zebrafish embryos injected with ABE8e+tp53, ABE-Umax-ex1+tp53, ABE-Umax-ex2+tp53, and their respective mRNA only. Error bars indicate mean ± s.d. (n = 3 biological replicates). Two-tailed paired t-tests were performed (with P-values marked).

(d) Representative jitter plots displaying the RNA A-to-I conversion frequencies at the transcriptome level in zebrafish embryos injected with ABE8e+tp53, ABE-Umax-ex1+tp53, ABE-Umax-ex2+tp53, and their respective mRNA only. Error bars indicate mean ± s.d. (n = 3 biological replicates). All box plots include the median line; the box denotes the interquartile range (IQR), whiskers denote the rest of the data distribution, and outliers are denoted by points greater than ±1.5 × IQR. Two-tailed paired t-tests were performed (with P-values marked).

All source data are provided as a Source Data file.

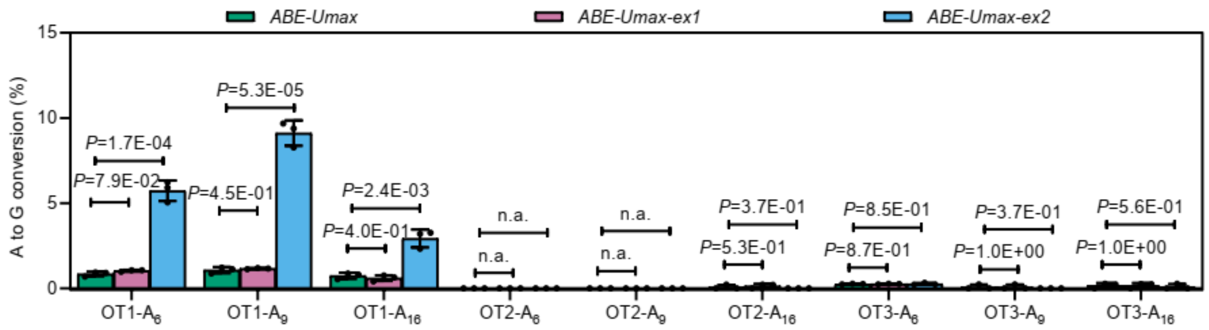

Supplementary Figure 10. DNA Off-Target Analysis of ABE-Umax-ex1 and ABE-Umax-ex2 in Zebrafish at the *Tyr*-g4 Locus

DNA off-target analysis comparing ABE-Umax, ABE-Umax-ex1, and ABE-Umax-ex2 at the Tyr-g4 locus. Editing efficiencies are shown. Error bars indicate mean ± s.d. (n = 3 biological replicates). Two-tailed paired t-tests were performed (with P-values marked). Source data are provided as a Source Data file.

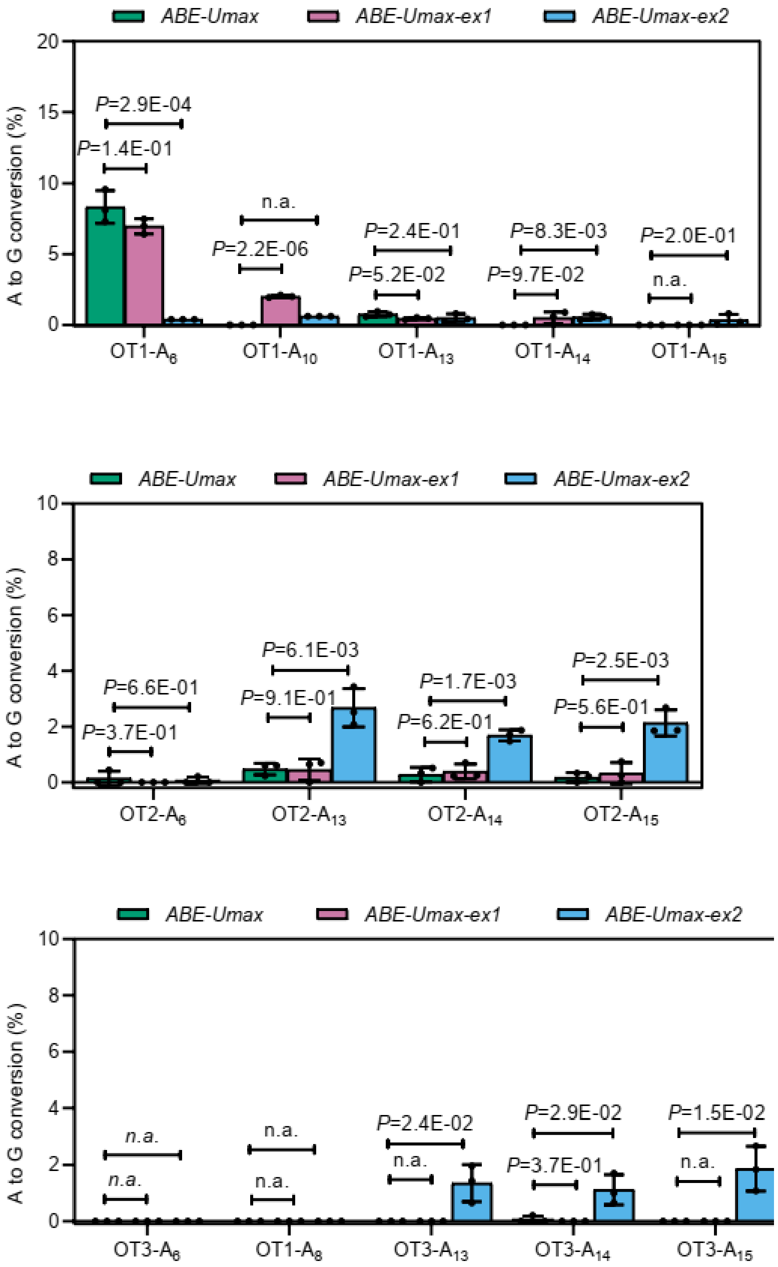

Supplementary Figure 11. DNA Off-target analysis of ABE-Umax-ex1 and ABE-Umax-ex2 in zebrafish at *tp53* locus.

Analysis of off-target DNA editing by ABE-Umax, ABE-Umax-ex1, and ABE-Umax-ex2 at the *tp53* locus in zebrafish. Editing efficiencies are shown. Error bars indicate mean ± standard deviation (n=3 biological replicates). Data were analyzed by two-tailed paired t-test (P values are shown). Source data are provided as a Source Data file.

a

Sample	Off-target site	Mismatched bases (bp)	Genome location	Sequence
tyr-g4	1	2	5:45224146-45224168	GCTgCAGCAcCTTTGAGCAG TGG
	2	2	23:37738775-37738797	GCTtCAGCATCTTTGAGaAG AAC
	3	2	3:45409654-45409676	tCTCCAGCATCTTTGAGCAt TTT

b

Sample	Off-target site	Mismatched bases (bp)	Genome location	Sequence
tp53	1	1	24:26468287-26468309	CAATCAGgGAGCAAATTACA GGG
	2	2	19:8750173-8750195	CAATCAGgGtGCAAATTACA GGG
	3	2	17:38879457-38879479	tAATCAGCaAGCAAATTACA ACC

c

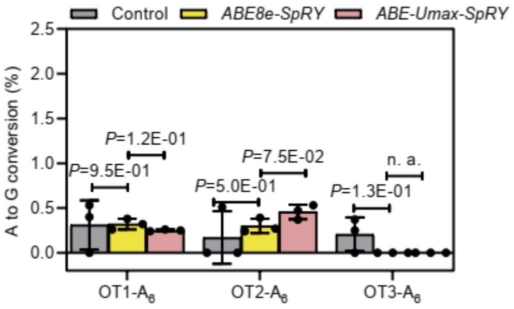

d

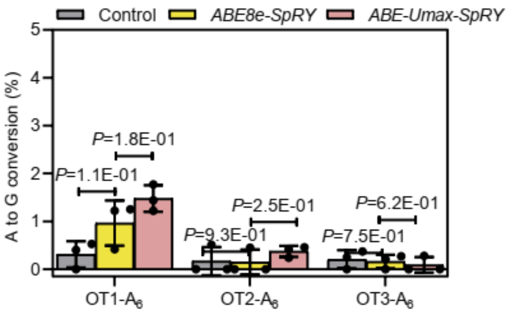

Supplementary Figure 12. DNA Off-target Analysis of ABE-Umax-SpRY in Zebrafish.

(a, b) The top three most likely off-target sites for ABE-Umax-SpRY at the *tyr-g4* and *tp53* loci are shown. Mismatched bases in potential off-target sites are indicated in lowercase, with the potentially editable adenine (A) highlighted in red.

(c) Comparison of DNA off-target editing efficiencies between ABE-Umax-SpRY and ABE-8e-SpRY at the *tyr-g4* locus. Error bars represent mean ± standard deviation (s.d.) (n = 3 biological replicates). Two-tailed paired t-tests were performed (P values indicated).

(d) Comparison of DNA off-target editing efficiencies between ABE-Umax-SpRY and ABE-8e-SpRY at the *tp53* locus. Error bars represent mean ± standard deviation (s.d.) (n = 3 biological replicates). Two-tailed paired t-tests were performed (P values indicated).

Source data are provided as a Source Data file.

Gene	Code name	Target sequence+**PAM**	Position	Mutation in human	Mutation in zebrafish	ClinVar inforamtion	Pathogenicity prediction
myo7aa	*myo7aa* (Y414C)	TTTATGGCCGTTTGTTTGTG **TGG**	4	NM_000260.4 (*MYO7A*): c. 1208A>G (p. Tyr403Cys), Y403C	ENSDART00000174975.2 (*myo7aa*): c. 1241A>G, Y414C	Likely pathogenic	0.941
ush1c	*ush1c* (M1V)	gttcaccATGGAAAGAAAAG **TGG**	8	NM_153676.4 (*USH1C*): c. 1A>G (p. Met1Val), M1V	ENSDART00000121791.4 (*ush1c*): c. 1A>G, M1V	Likely pathogenic	0.939
spata5l1	*spata5l1* (Q313R	CGCGCAGCTGCTAACGCTAA **TGG**	6	NM_024063.3 (*SPATA5L1*): c. 971A>G (p. Gln324Arg), Q324R	ENSDART00000129459.3 (*spata5l1*): c. 938A>G, Q313R	NA	0.964
eprs1	*eprs1* (W1318R)	CACCACTGGTTGAGTTTGAT **TGG**	5	NM_004446.3 (*EPRS1*): c. 3439T>C (p. Trp1147Arg), W1147R	ENSDART00000075743.7 (*eprs1*): c. 3952T>C, W1318R	NA	0.928
cog1	*cog1* (Q49R)	TCCGGCAGATGGTCGGTGAG **CGG**	7	NM_018714.3 (*COG1*): c. 149A>G (p. Gln50Arg), Q50R	ENSDART00000167896.3 (*cog1*): c. 146A>G, Q49R	NA	0.879
adam22	*adam22* (Q95R)	AGCTTCCAGGTGCAAGCTTT **TGG**	8	NM_001324418.2 (*ADAM22*): c. 275A>G (p. Gln92Arg), Q92R	ENSDART00000125590.4 (*adam22*): c. 284A>G, Q95R	NA	0.177
adam22	*adam22* (S869P)	CTGTGGAGTTGGAGCGAGCA **CGG**	7	NM_021721.3 (*ADAM22*): c. 2569T>C (p. Ser869Pro), S857P	ENSDART00000125590.4 (*adam22*): c. 2605T>C, S869P	NA	0.612

Supplementary Figure 13. Summary of predicted information for variants of uncertain significance (VUS) tested in this study. The targeted adenine (A) at each site is highlighted in red.

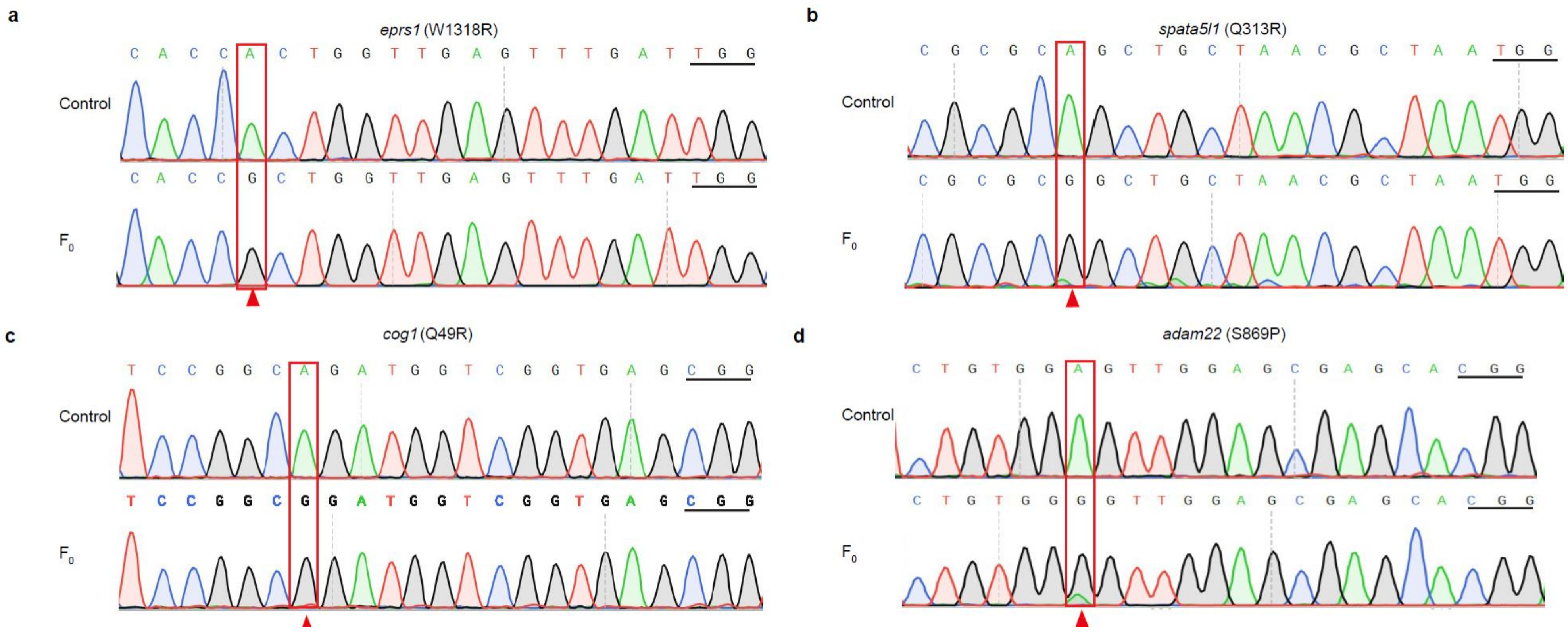

Supplementary Figure 14. Sanger sequencing results of A→G editing on four diffwerent locus. (a) *eprs1* (W1318R), (b)*spata5l1* (Q313R), (c)*cog1* (Q49R) and (d) *adam22* (S869P) F_0 embryos induced by ABE-Umax. The nucleotide substitutions are indicated by a red arrowhead in the Sanger sequencing chromatograms.
