# Supplementary material for: ABE-ultramax for high-efficiency biallelic adenine base editing in zebrafish
Source: Nat Commun. 2024 Jul 4;15:5613. doi: 10.1038/s41467-024-49943-1 (PMC11224239; doi:10.1038/s41467-024-49943-1)
Supplement: Supplementary file 3 — Description Of Additional Supplementary File [file 41467_2024_49943_MOESM3_ESM.pdf]

Description of Additional supplementary file

Supplementary Data 1. Germline targeting efficiency of base edited targets by ABE-Umax.

Supplementary Data 2. Germline targeting efficiency and germline transmission rate of ABE-Umax-nanos1-induced base editing.

Supplementary Data 3. Primers for constructing plasmids.

Supplementary Data 4. All target sites and detection primers.
